# The association between non-high-density lipoprotein cholesterol to high-density lipoprotein cholesterol ratio (NHHR) and kidney stones: a cross-sectional study

**DOI:** 10.1186/s12944-024-02089-x

**Published:** 2024-04-13

**Authors:** Hujian Hong, Yijiang He, Zhiqiang Gong, Jilong Feng, Yanli Qu

**Affiliations:** 1 Department of Radiation Oncology, Cancer Hospital of China Medical University, Liaoning Cancer Hospital & Institute, Cancer Hospital of Dalian University of Technology, No.44 Xiaoheyan Road, Dadong District, Shenyang, 110042 Liaoning China; 2 School of Graduate, Dalian Medical University, Dalian, 116044 Liaoning China; 3 Department of Radiotherapy, Shenyang Fifth People’s Hospital, No.188 Xingshun Street, Tiexi District, Shenyang, 110023 Liaoning China

**Keywords:** NHHR, Lipid ratio, Kidney stones, NHANES, Cross-sectional study

## Abstract

**Background:**

The relationship between the NHHR and kidney stone risk remains unknown. The purpose of this study was to evaluate the association between adult NHHR and kidney stone occurrence in USA.

**Methods:**

This study used a variety of statistical techniques such as threshold effects, subgroup analysis, smooth curve fitting, multivariate logistic regression, and data from the National Health and Nutrition Examination Survey (NHANES) from 2007 to 2014. We aimed to clarify the relationship between the NHHR and kidney stone risk.

**Results:**

The average age of the 21,058 individuals in this research was 49.70 ± 17.64 years. The mean NHHR was 3.00 ± 1.47, and the overall prevalence of kidney stone occurrence was 9.05%. The prevalence within the quartile ranges (Q1–Q4) was 7.01%, 8.71%, 9.98%, and 10.49%, respectively. The overall average recurrence rate of kidney stones was 3.05%, demonstrating a significant increase with increasing NHHR (Q1: 1.92%, Q2: 2.92%, Q3: 3.35%, Q4: 4.00%, *P* < 0.01). The occurrence of kidney stones increased by 4% (95% CI: 1.00-1.08, *P* = 0.0373) and the chance of recurrence increased by 9% (95% CI: 1.03–1.14, *P* < 0.01) with each unit increase in NHHR. The interaction analysis results demonstrated that the relationship between the NHHR and the risk of kidney stones was not significantly impacted by the following factors: sex, body mass index, poverty income ratio, diabetes, or hypertension. Curve fitting and threshold effect analysis also demonstrated a non-linear association, with a breakpoint found at 3.17, between the NHHR and the risk of kidney stones.

**Conclusions:**

In adults in the USA, there is a substantial correlation between elevated NHHR levels and a higher probability of kidney stones developing and recurring. Timely intervention and management of NHHR may effectively mitigate the occurrence and recurrence of kidney stones.

## Introduction

Nephrolithiasis is a prevalent malady of the urogenital system and is attributed to excessive mineral saturation in urine, giving rise to crystalline formations that subsequently precipitate within the renal pelvis and calyces [1]. According to epidemiological statistics, there are 114–720 cases per 100,000 people in Italy, Japan, Germany, Scotland, Spain, Sweden, and the USA, with prevalence rates ranging from 1.7 to 14.8% [2]. Moreover, its incidence rate has increased sharply over the past three decades [3]. Furthermore, the recurrence rate of nephrolithiasis is notably elevated, with an estimated annual recurrence rate ranging from 10 to 23%, escalating to 50% within 5–10 years and reaching 75% within two decades [4, 5].

Nephrolithiasis can cause renal colic, haematuria, obstructive hydronephrosis, and impaired renal function. In severe cases, complications, including infections, may occur with potentially life-threatening implications [6–8]. Obesity [9], diabetes [10, 11], hypertension [12], and metabolic syndrome [13]are acknowledged as pivotal risk factors. The current study underscores the correlation between dyslipidaemia and nephrolithiasis. A nationwide survey conducted by Kohjimoto et al. established an association between dyslipidaemia and an increased prevalence of stone recurrence or multiplicity. Dyslipidaemia may exacerbate the risk of nephrolithiasis through mechanisms such as insulin resistance, inflammatory responses, and oxidative stress [14]. Additionally, studies have suggested a significant elevation in uric acid levels and decrease in urine pH among individuals with metabolic syndrome, which correlates with an increased occurrence of uric acid stones. Within this demographic, reduced levels of high density lipoprotein (HDL) and increased levels of triglycerides have been linked to an increased incidence of uric acid stones [15]. A newly identified risk indicator for atherosclerosis is the ratio of non-high density lipoprotein cholesterol to high density lipoprotein cholesterol (NHHR) [16]. Recent investigations have indicated that the NHHR can independently determine the risk of metabolic syndrome, chronic kidney disease, and nonalcoholic fatty liver disease (NAFLD) [17–20]. However, the relationship between the NHHR and nephrolithiasis has not yet been explored. Thus, by leveraging NHANES data from 2007 to 2014, this study sought to elucidate the correlation between NHHR and the likelihood of developing nephrolithiasis. This study hypothesised that high NHHR increases the likelihood of nephritis, and exploring the association between lipid metabolism and kidney stones is expected to fill a knowledge gap in the research field. Simultaneously, we opened up a new area of research to explore the potential application of the NHHR in predicting the outcome of kidney stones.

## Methods

### Data source

National Health and Nutrition Examination Survey (NHANES) is a comprehensive survey addressing various ethnic groups and health-related issues in the USA. It endeavours to amass information pertaining to the health, nutritional, and sociological aspects of the American population. The program ensures that all participants have provided their given permission by conducting health and nutrition evaluations every two years, which are examined and authorized by the National Centre for Health Statistics Research Ethics Review Board. The database contains structured questionnaires, physical examinations, and laboratory tests. Requisite data were procured from the official NHANES website.

### Study population

**Fig. 1:**
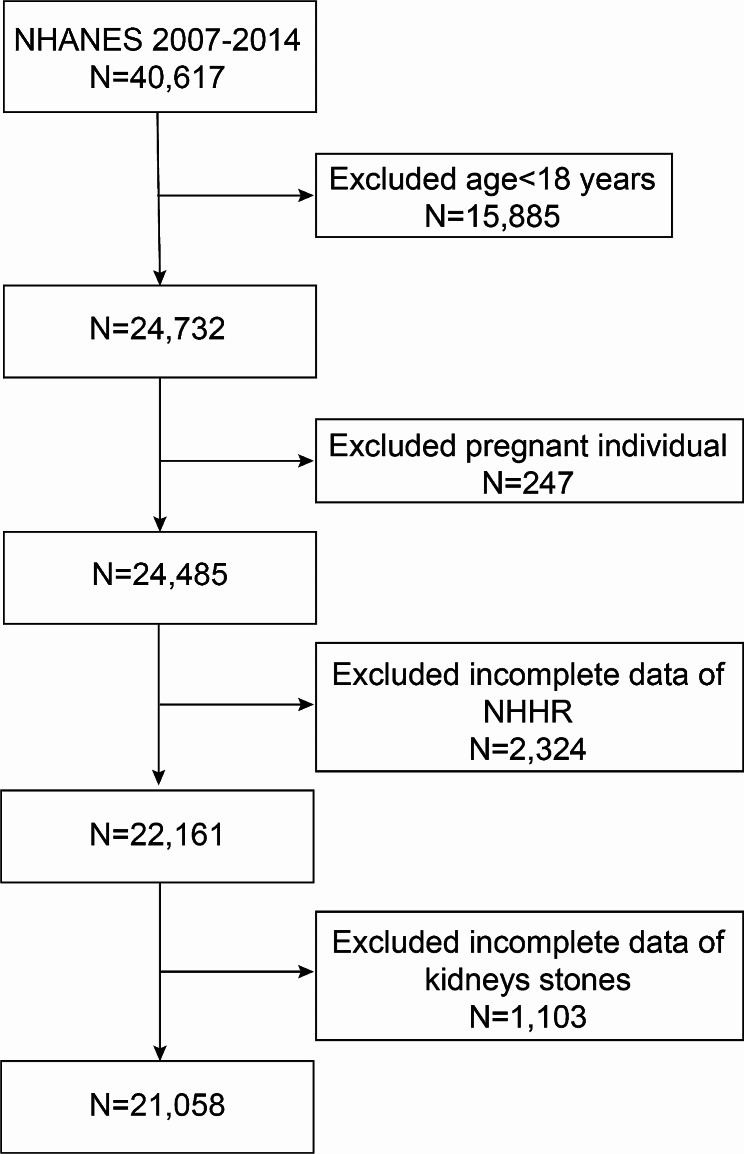
The flowchart depicting sample selection for the National Health and Nutrition Examination Survey (NHANES) from 2007 to 2014

This study meticulously curated data from four NHANES survey cycles from 2007 to 2014. Initially, the cohort comprised of 40,617 individuals. Through a methodological screening process, certain demographic characteristics were excluded as follows: individuals under the age of 18 years (15,885 individuals), pregnant women (247 individuals), those lacking NHHR data (2,324 individuals), and those who did not provide information on kidney stones (1,103 individuals). Following this rigorous selection process, 21,058 individuals met the inclusion criteria (Fig. 1).

### Exposure definition

The focal exposure variable in this study was NHHR, which denotes the ratio of non-high-density lipoprotein cholesterol to high-density lipoprotein cholesterol [21]. The computation of non-high-density lipoprotein cholesterol involves subtracting high-density lipoprotein cholesterol from total cholesterol (TC). Stratifying participants based on their NHHR values, this study categorised them into four tiers for analytical convenience.

### Outcome definition

Nephrolithiasis occurrence and recurrence rates were regarded as outcome measures in this study. These metrics were ascertained through a health status questionnaire in the NHANES, relying on the participants’ self-reports during personal interviews regarding two specific renal health enquiries (KIQ026 and KID028). These queries respectively enquire, ‘Have you ever suffered from nephrolithiasis?’ and ‘How many instances of nephrolithiasis did you experience?’ Participants were required to choose ‘yes’ or ‘no’ as their responses. An affirmative response was defined as a history of nephrolithiasis. If there were two or more documented instances of affliction, it was classified as a nephrolithiasis recurrence. Prior research has substantiated the heightened accuracy of nephrolithiasis information obtained using self-reporting methods [22].

### Covariates

Drawing on prior research, this study extracted a myriad of covariates from the NHANES database, encompassing dimensions such as demographics, dietary habits, examinations, laboratory assays, and questionnaire surveys. Numerous categorical factors were included in these covariates, such as body mass index (BMI), age, sex, ethnicity, marital status, educational level, poverty income ratio (PIR), vigorous and moderate physical activities, daily alcohol consumption (defined as a minimum intake of four alcoholic beverages per day), smoking habits (defined as at least 100 cigarettes smoked throughout an individual’s lifetime), and the presence of hypertension and diabetes. Notably, the PIR is stratified into three tiers: ‘1’, ‘1–3’, and ‘3’ or above. Similarly, the BMI is divided into ranges of less than 25, 25–29.9, and more than 30 kg/m², which represent normal weight, overweight, and obesity, respectively. For continuous variables, our considerations were extended to the levels of TC and high-density lipoprotein cholesterol (HDL-C).

### Statistical analysis

In accordance with the guidelines issued by the Centres for Disease Control and Prevention (CDC) (https://wwwn.cdc.gov/nchs/nhanes/tutorials/default.aspx), this study conducted refined statistical analyses. The specific procedures were as follows: initially, quartile division of the NHHR was performed, with the lowest quartile (Q1) serving as the reference group. Frequencies and percentages are used to portray categorical data, and the standard deviation, or mean, is used to describe continuous variables. The association between the NHHR and the occurrence and recurrence rates of kidney stones was investigated using a multivariate logistic regression model. Model 2 contained sex, age, and ethnicity adjustments, and Model 3 added more adjustments for BMI, education level, marital status, PIR, alcohol and smoking habits, diabetes, hypertension, and vigorous and moderate physical activity in addition to the adjustments for sex, age, and ethnicity. Step three involved testing the threshold effect of the NHHR on the occurrence rate of kidney stones using a segmented linear regression model and examining the nonlinear connection between the NHHR and the occurrence and recurrence rates of kidney stones using a smooth curve-fitting approach. Finally, an in-depth exploration of the potential differences among different populations was conducted through subgroup analysis and interaction testing. EmpowerStats (version 2.0) and R software (version 4.1.3) were utilized for each of the statistical evaluations, with a significance level of *P* below 0.05.

## Results

### Baseline characteristics of participants

Table 1 delineates the baseline characteristics of the participants selected from the NHANES between 2007 and 2014, stratified by NHHR quartiles. The study included 21,058 participants, with a sex distribution of 49.18% males and 50.82% females. The mean age was 49.70 years, with a standard deviation of 17.64 years. The quartile ranges for NHHR were 0.45–1.98, 1.98–2.72, 2.72–3.71, and 3.71–24.3. The overall occurrence rate of kidney stones averaged 9.05%, with the rates for each quartile (Q1 to Q4) being 7.01%, 8.71%, 9.98%, and 10.49%. The overall average recurrence rate of kidney stones was 3.05%, exhibiting a significant increase with increasing NHHR (Q1:1.92%; Q2:2.92%; Q3:3.35%; Q4:4.00%; *P* < 0.01).

**Table 1:** Based on the baseline characteristics of the study population ascertained by NHANES from 2007 to 2014

Characteristic	Total	Q1 (0.45–1.98)	Q2 (1.98–2.72)	Q3 (2.72–3.71)	Q4 (3.71–24.3)	*P*-value
N	21,058	5261	5268	5258	5271	
Age (years)	49.70 ± 17.64	49.48 ± 19.31	50.50 ± 18.28	49.86 ± 17.08	48.95 ± 15.63	< 0.001
Sex (%)						< 0.001
Male	10,357 (49.18%)	1928 (36.65%)	2248 (42.67%)	2841 (54.03%)	3340 (63.37%)	
Female	10,701 (50.82%)	3333 (63.35%)	3020 (57.33%)	2417 (45.97%)	1931 (36.63%)	
Race (%)						< 0.001
Mexican American	3156 (14.99%)	561 (10.66%)	705 (13.38%)	887 (16.87%)	1003 (19.03%)	
Other Hispanic	2137 (10.15%)	410 (7.79%)	517 (9.81%)	576 (10.95%)	634 (12.03%)	
Non-Hispanic White	9426 (44.76%)	2341 (44.50%)	2353 (44.67%)	2316 (44.05%)	2416 (45.84%)	
Non-Hispanic Black	4237 (20.12%)	1382 (26.27%)	1142 (21.68%)	975 (18.54%)	738 (14.00%)	
Other Races	2102 (9.98%)	567 (10.78%)	551 (10.46%)	504 (9.59%)	480 (9.11%)	
Marital status (%)						< 0.001
Married	10,891 (51.72%)	2435 (46.28%)	2642 (50.15%)	2868 (54.55%)	2946 (55.89%)	
Single	8607 (40.87%)	2479 (47.12%)	2226 (42.26%)	2021 (38.44%)	1881 (35.69%)	
with partner	1560 (7.41%)	347 (6.60%)	400 (7.59%)	369 (7.02%)	444 (8.42%)	
Education level (%)						< 0.001
Below high school	2317 (11.00%)	424 (8.06%)	541 (10.27%)	617 (11.73%)	735 (13.94%)	
High school	3195 (15.17%)	738 (14.03%)	729 (13.84%)	809 (15.39%)	919 (17.44%)	
Above high school	15,546 (73.82%)	4099 (77.91%)	3998 (75.89%)	3832 (72.88%)	3617 (68.62%)	
PIR (%)						< 0.001
< 1	4299 (22.31%)	1031 (21.36%)	1017 (21.13%)	1045 (21.76%)	1206 (24.99%)	
1–3	7979 (41.41%)	1865 (38.64%)	1919 (39.87%)	2097 (43.66%)	2098 (43.48%)	
> 3	6989 (36.27%)	1930 (39.99%)	1877 (39.00%)	1661 (34.58%)	1521 (31.52%)	
BMI category (%)						< 0.001
Normal weight	6147 (29.56%)	2623 (50.37%)	1679 (32.31%)	1135 (21.85%)	710 (13.66%)	
Overweight	7001 (33.66%)	1503 (28.86%)	1739 (33.46%)	1849 (35.60%)	1910 (36.74%)	
Obese	7649 (36.78%)	1081 (20.76%)	1779 (34.23%)	2210 (42.55%)	2579 (49.61%)	
Vigorous activity (%)						< 0.001
Yes	3799 (18.04%)	787 (14.96%)	895 (16.99%)	988 (18.79%)	1129 (21.42%)	
No	17,259 (81.96%)	4474 (85.04%)	4373 (83.01%)	4270 (81.21%)	4142 (78.58%)	
Moderate activity (%)						< 0.001
Yes	7279 (34.57%)	1708 (32.47%)	1755 (33.31%)	1890 (35.95%)	1926 (36.54%)	
No	13,779 (65.43%)	3553 (67.53%)	3513 (66.69%)	3368 (64.05%)	3345 (63.46%)	
Alcohol habit (%)						< 0.001
Yes	2865 (17.42%)	599 (14.70%)	642 (15.77%)	729 (17.82%)	895 (21.26%)	
No	13,582 (82.58%)	3476 (85.30%)	3428 (84.23%)	3363 (82.18%)	3315 (78.74%)	
Smoking habit (%)						< 0.001
Yes	9541 (45.31%)	2191 (41.65%)	2281 (43.30%)	2369 (45.06%)	2700 (51.22%)	
No	11,517 (54.69%)	3070 (58.35%)	2987 (56.70%)	2889 (54.94%)	2571 (48.78%)	
Hypertension (%)						< 0.001
Yes	7596 (36.07%)	1786 (33.95%)	1877 (35.63%)	1963 (37.33%)	1970 (37.37%)	
No	13,462 (63.93%)	3475 (66.05%)	3391 (64.37%)	3295 (62.67%)	3301 (62.63%)	
Diabetes (%)						0.138
Yes	2611 (12.40%)	619 (11.77%)	637 (12.09%)	660 (12.55%)	695 (13.19%)	
No	18,447 (87.60%)	4642 (88.23%)	4631 (87.91%)	4598 (87.45%)	4576 (86.81%)	
TC, mmol/L	5.01 ± 1.08	4.42 ± 0.89	4.76 ± 0.90	5.08 ± 0.89	5.77 ± 1.12	< 0.001
HDL-C, mmol/L	1.35 ± 0.41	1.77 ± 0.42	1.43 ± 0.28	1.22 ± 0.22	0.99 ± 0.20	< 0.001
NHHR	3.00 ± 1.47	1.53 ± 0.32	2.34 ± 0.21	3.18 ± 0.28	4.95 ± 1.41	< 0.001
Nephrolithiasis (%)						< 0.001
Yes	1906 (9.05%)	369 (7.01%)	459 (8.71%)	525 (9.98%)	553 (10.49%)	
No	19,152 (90.95%)	4892 (92.99%)	4809 (91.29%)	4733 (90.02%)	4718 (89.51%)	
Nephrolithiasis recurrence (%)						0.009
Yes	642 (3.05%)	101 (1.92%)	154 (2.92%)	176 (3.35%)	211 (4.00%)	
No	20,416 (96.95%)	5160 (98.08%)	5114 (97.08%)	5082 (96.65%)	5060 (96.00%)	

### Associations between the NHHR and kidney stones

Regarding the incidence of kidney stones, this study demonstrated a positive link between the likelihood of kidney stone occurrence and an increase in NHHR. After making all necessary modifications, the chance of developing kidney stones rose by 4% (95% CI: 1.00-1.08, *P* = 0.0373) for every incremental unit rise in NHHR. In addition, using the NHHR as a stratified variable (quartiles) for further analysis, individuals in the highest quartile (Q4) had a 1.28-fold higher risk of kidney stone incidence in a fully corrected model (95% CI: 1.07–1.52, *P* = 0.0058) than those in the lowest quartile (Q1). This strengthens the favorable link that has been seen throughout time between higher NHHR and kidney stone risk.

The study also shows a correlation between elevated NHHR and a higher chance of kidney stone recurrence (Model 1: OR = 1.14, 95% CI: 1.10–1.19, *P* < 0.01; Model 2: OR = 1.12, 95% CI: 1.07–1.17, *P* < 0.01; Model 3: OR = 1.09, 95% CI: 1.03–1.14, *P* < 0.01). A consistent positive connection was found between the risk of kidney stone recurrence and NHHR rise in the fully calibrated Model 3, with data showing that for every unit increase in NHHR, the likelihood of recurrence rose by 9% (Table 2).

**Table 2:** Association of NHHR with kidney stone and a recurrence of passing kidney stones

Exposure	Model 1	Model 2	Model 3
	OR(95%CI), *P*-value	OR(95%CI), *P*-value	OR(95%CI), *P*-value
Nephrolithiasis			
NHHR	1.08 (1.05, 1.12) < 0.0001	1.07 (1.04, 1.11) < 0.0001	1.04 (1.00, 1.08) 0.0373
Categories			
Q1	1.0	1.0	1.0
Q2	1.27 (1.10, 1.46) 0.0012	1.22 (1.05, 1.41) 0.0074	1.15 (0.97, 1.37) 0.1132
Q3	1.47 (1.28, 1.69) < 0.0001	1.40 (1.22, 1.62) < 0.0001	1.25 (1.05, 1.49) 0.0104
Q4	1.55 (1.35, 1.78) < 0.0001	1.47 (1.27, 1.69) < 0.0001	1.28 (1.07, 1.52) 0.0058
P for trend	1.14 (1.10, 1.19) < 0.0001	1.13 (1.08, 1.18) < 0.0001	1.08 (1.02, 1.14) 0.0086
Nephrolithiasis recurrence			
NHHR	1.14 (1.10, 1.19) < 0.01	1.12 (1.07, 1.17) < 0.01	1.09 (1.03, 1.14) < 0.01
Categories			
Q1	1.0	1.0	1.0
Q2	1.54 (1.19, 1.98) 0.0009	1.46 (1.13, 1.89) 0.0037	1.26 (0.93, 1.70) 0.1380
Q3	1.77 (1.38, 2.27) < 0.0001	1.63 (1.27, 2.09) 0.0001	1.34 (1.00, 1.81) 0.0527
Q4	2.13 (1.68, 2.71) < 0.0001	1.90 (1.49, 2.44) < 0.0001	1.58 (1.18, 2.12) 0.0024
*P* for trend	1.24 (1.16, 1.33) < 0.0001	1.20 (1.12, 1.29) < 0.0001	1.15 (1.05, 1.25) 0.0026

In the analysis, NHHR was transformed from a continuous variable into a categorical variable using quartiles

OR, odds ratio; 95% CI, 95% confidence interval

Model 1 did not incorporate any variable adjustments

Model 2 adjusted for sex, age, and race

Model 3 extended these adjustments to include a more comprehensive set of variables: sex, age, race, educational level, marital status, Poverty Income Ratio (PIR), Body Mass Index (BMI), vigorous activity, moderate activity, hypertension, diabetes, alcohol and smoking habits

### Nonlinear association between the NHHR and Kidney Stones

**Fig. 2:**
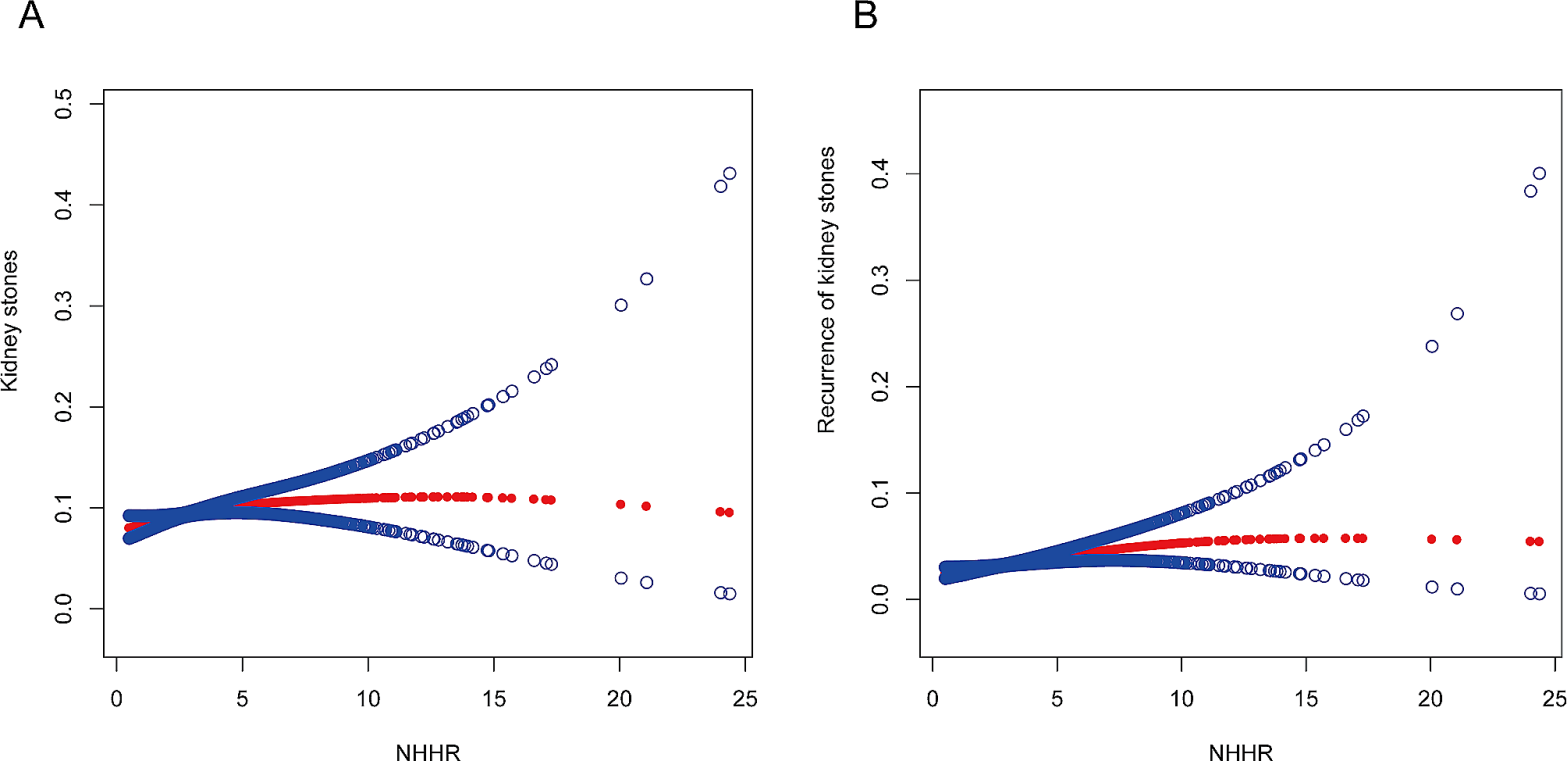
(**A**) The correlation between NHHR and kidney stones. (**B**) The association between NHHR and the recurrence of kidney stones. The red solid line signifies the smooth curve fit between variables. The blue band represents the 95% confidence interval derived from the fit

This study investigated the nonlinear relationship between the NHHR and the risk of kidney stones, as seen in Fig. 2. Through meticulous analysis of smooth curves, this study revealed a nonlinear association between the occurrence of kidney stones and the NHHR (Part A). In the correlation analysis of kidney stone recurrence, a similar nonlinear pattern is observed (Part B). On contrasting standard linear models with biphasic linear models, the research findings indicated that in the likelihood ratio test, the *P*-value for the association between NHHR and kidney stone recurrence was 0.07, whereas the *P*-value for NHHR and the risk of kidney stone occurrence was significantly < 0.05. By employing biphasic linear models and recursive algorithms, the study identifies an inflection point at an NHHR value 3.17 (refer to Table 3). When NHHR is below 3.17, the chance of developing kidney stones increases by 15% (OR: 1.15, 95% CI: 1.04–1.27) for every additional unit of NHHR. Conversely, when the NHHR exceeds 3.17, no significant change in the relative risk of kidney stones was observed.

**Table 3:** Utilize the two-segment piecewise linear regression model for the analysis of threshold effects between NHHR and nephrolithiasis

Adjusted		
	OR (95% CI)	P value
Nephrolithiasis		
Fitting by standard linear model	1.04 (1.00, 1.08)	0.037
Fitting by two-piecewise linear model		
<3.17	1.15 (1.04, 1.27)	0.0076
>3.17	1.00 (0.95, 1.06)	0.9566
Log-likelihood ratio	0.039	
Nephrolithiasis recurrence		
Fitting by standard linear model	1.09 (1.03, 1.14)	0.0025
Fitting by two-piecewise linear		
<5.43	1.15 (1.06, 1.25)	0.0009
>5.43	0.99 (0.87, 1.13)	0.8860
Log-likelihood ratio	0.070	

Adjusted for sex, age, race, educational level, marital status, Poverty Income Ratio (PIR), Body Mass Index (BMI), vigorous activity, moderate activity, hypertension, diabetes, alcohol and smoking habits

### Subgroup analysis

This study investigated whether kidney stones and NHHR status are consistently associated in the general population using subgroup analyses and interaction testing. It seeks to identify possible differences in particular demographic scenarios according to BMI, PIR, sex, diabetes, and hypertension, among others. Table 4 presents the research findings, which indicate a positive association consistently across sex, BMI, PIR, diabetes, and hypertension subgroups with kidney stone occurrence. This finding suggests that the correlation applies to a wide variety of demographic situations.

**Table 4:** Subgroup analysis

NHHR	Nephrolithiasis		Nephrolithiasis recurrence	
	OR(95%CI), *P*-value	*P* for interaction	OR(95%CI), *P*-value	*P* for interaction
Sex		0.4571		0.4464
Male	1.04 (0.99, 1.09) 0.1221		1.08 (1.01, 1.16) 0.0277	
Female	1.07 (1.01, 1.14) 0.0325		1.13 (1.03, 1.23) 0.0063	
BMI		0.5341		0.6919
Normal weight	1.03 (0.92, 1.14) 0.6090		1.06 (0.89, 1.27) 0.4856	
Overweight	1.01 (0.95, 1.08) 0.6755		1.05 (0.95, 1.16) 0.3062	
Obese	1.06 (1.01, 1.11) 0.0223		1.10 (1.03, 1.18) 0.0037	
PIR		0.2518		0.5233
< 1	1.00 (0.93, 1.08) 0.9755		1.11 (1.02, 1.22) 0.0185	
1–3	1.03 (0.98, 1.09) 0.2798		1.05 (0.96, 1.14) 0.2803	
> 3	1.09 (1.02, 1.16) 0.0152		1.12 (1.00, 1.24) 0.0458	
Diabetes		0.7834		0.5237
Yes	1.05 (0.97, 1.13) 0.2285		1.05 (0.94, 1.18) 0.3913	
No	1.04 (0.99, 1.08) 0.1053		1.09 (1.03, 1.16) 0.0031	
Hypertension		0.8378		0.3268
Yes	1.03 (0.97, 1.08) 0.3320		1.05 (0.97, 1.13) 0.2459	
No	1.03 (0.98, 1.09) 0.1947		1.10 (1.03, 1.18) 0.0060	

## Discussion

The purpose of this study was to investigate the possible relationship between kidney stones and NHHR. The analysis of 21,058 participants revealed a connection between an elevated NHHR and an increased risk of both the initial occurrence and recurrence of kidney stones. This correlation remained consistent when the subgroups were stratified based on sex, BMI, PIR, diabetes, and hypertension. Further smoothing curve fitting and threshold effect analysis indicated a nonlinear relationship between the NHHR and kidney stones, with a turning point identified at 3.17. Before this inflection point, an increase in the NHHR correlated positively with the risk of kidney stone occurrence, whereas beyond this point, the correlation was not statistically significant.

This study provides a preliminary investigation into the association between the NHHR and kidney stones. The mounting evidence acknowledges NHHR as a precise indicator of lipid-related disease risks [18, 23]. Despite the lack of direct research exploring the link between kidney stones and lipid metabolism, extensive studies have revealed associations between kidney stones and various lipid-related factors. An 8-year prospective study found a significant increase in kidney stone risk with elevated triglyceride levels [24]. Comparative studies have indicated higher serum triglyceride and lower HDL-C levels in patients with kidney stones [25–30]. A retrospective analysis of 2,442 kidney stone patients showed an association between lipid levels and changes in urine composition [31]. Furthermore, dyslipidemia is linked to an increased prevalence of stone recurrence or multiplicity [14]. In individuals with metabolic syndrome, elevated triglyceride and low HDL levels are closely associated with an increased incidence of uric acid stones [15]. Fundamentally, kidney stones consist of crystals and organic matrices, with the matrix containing various lipids that facilitate crystal nucleation and drive stone formation [32–36].

NHHR, which is recognized as a novel lipid indicator of atherosclerosis [37], serves as an independent risk factor for atherosclerotic plaques and is a crucial lipid marker for preventing plaque formation [21]. NHHR has demonstrated diagnostic value beyond traditional lipid markers in predicting metabolic syndrome, insulin resistance [19, 38], and NAFLD [17]. The close association of the NHHR with various diseases validates its effectiveness as a lipid management tool. This study also identified a potential positive correlation between the NHHR and the occurrence and recurrence of kidney stones. Therefore, the NHHR is a useful tool for determining how lipid metabolism affects kidney stone occurrence and probable recurrence.

## Study strengths and limitations

There were several significant research strengths in this study. It started with a nationwide poll of individuals in the United States. Second, meticulously adjusting for confounding variables ensured the credibility and generalizability of the research outcomes. Finally, employing smoothing curve fitting and bilinear regression models, it explores the nonlinear relationship between NHHR and kidney stones.

However, this study had some limitations. First, the diagnosis of kidney stones relied on self-reporting by participants, introducing subjectivity and inevitable recall bias. Second, despite adjusting for numerous confounding factors based on prior research, the potential impact of unmeasured or unknown confounding factors on the study results cannot be completely eliminated. The cross-sectional design of this investigation precluded the establishment of a causal link between kidney stones and the NHANES. Finally, due to the data design of the NHANES database, this study implemented some exclusion criteria that may have been subject to selection bias.

## Conclusion

The findings of this study showed that the NHHR and the risk of kidney stones and their recurrence were positively correlated. The potential prognostic value of the NHHR for kidney stone outcomes was another new area investigated in this study. Controlling NHHR and mitigating the risk of kidney stones have significant clinical implications. However, further prospective clinical trials are required to confirm the potential role of lipids in kidney disease.

## Data Availability

No datasets were generated or analysed during the current study.

## Abbreviations

NHANES: National Health and Nutrition Examination Survey
NHHR: Non-high-density lipoprotein cholesterol to high-density lipoprotein cholesterol ratio
BMI: Body mass index
PIR: Poverty income ratio
TC: Total cholesterol
LDL-C: Low-density lipoprotein cholesterol
HDL-C: High-density lipoprotein cholesterol
